# Mixed Method Evaluation of My Vital Cycles^®^: A Holistic School-Based Ovulatory Menstrual Health Literacy Program

**DOI:** 10.3390/ijerph20115964

**Published:** 2023-05-26

**Authors:** Felicity Roux, HuiJun Chih, Jacqueline Hendriks, Sharyn Burns

**Affiliations:** 1 Curtin Medical School, Curtin University, Bentley, WA 6102, Australia; 2 School of Population Health, Curtin University, Bentley, WA 6102, Australia

**Keywords:** health promoting school, strengths-based education, menstrual health literacy, ovulation, fertility, restorative reproductive medicine, mental health, body image, dysmenorrhea, abnormal uterine bleeding

## Abstract

There is a high prevalence of ovulatory menstrual (OM) dysfunctions among adolescents, and their menstrual health literacy is poor. The OM cycle can be used as a personal health monitor provided that the skills to understand it are correctly taught. My Vital Cycles^®^, a holistic school-based OM health literacy program, was trialed with a Grade 9 cohort in one single-sex school in Western Australia using the Health Promoting School framework. A validated OM health literacy questionnaire was administered pre- and post-program with 94 participants. Functional OM health literacy improved overall, with 15 out of 20 items showing improvement post-program (*p* < 0.05). In addition, 19 out of 53 items for interactive OM health literacy, and 18 out of 25 items for critical OM health literacy improved (*p* < 0.05). The improvement in mood concerns (*p* = 0.002) was unexpected. Thematic analysis of three focus groups of 18 girls revealed four themes of increasing comfort levels; finding the program informative; inclusion of non-teaching support such as healthcare professionals; and suggestions for future refinements. Overall, this Western Australian PhD project which developed and trialed My Vital Cycles^®^ improved OM health literacy and was positively received. Future research possibilities include understanding the program’s impact on mental health and further trials in co-educational settings; amongst different populations; and with extended post-program testing.

## 1. Introduction

The World Health Organization (WHO) defines health literacy as “the cognitive and social skills which determine the motivation and ability of individuals to gain access to, understand and use information in ways which promote and maintain good health” [1]. The ovulatory menstrual (OM) cycle is considered a “vital sign” of good health [2]. Skills in observing, interpreting, and managing the OM cycle would constitute a specific health literacy. Therefore, OM health literacy can be defined as firstly, the discipline of applying OM cycle knowledge and skills to maintain personal health by reference to ovulation which drives menstruation and with due cognizance of life stage and/or stressors, and secondly, confident engagement and active co-operation with healthcare providers to restore good reproductive health as needed [3].

There are seven reasons why OM health literacy is important. Firstly, OM cycles are a material reality for half of the world’s reproductively mature population. Moreover, OM cycles usually last for about 40 years [4]. Thirdly, as a biopsychosocial process [5], OM health embodies liminal milestones of menarche, fertility, and menopause. Additionally, the OM cycle has been considered a negative and stigmatizing experience [6,7], which risks cascading into shame around other healthy functions such as breastfeeding [8]. Fifthly, the OM cycle can act as a personal health monitor [2,9,10]. Relatedly, however, OM dysfunctions can occur. Examples of Australian adolescents include 93% self-reporting dysmenorrhea and 96% premenstrual symptoms [11]; up to 40% with heavy bleeding [12]; and 73% with mood disturbance [11]. Finally, OM health literacy makes possible the restoration of good health by co-operating with the OM cycle to resolve or manage OM dysfunctions using medications and/or surgery [13].

However, studies indicate that young women’s menstrual health literacy levels are low [14,15], with calls for improvements in menstrual health education [16,17].

Several menstrual health education initiatives are available in Australia. Period Talk™ offers lesson plans for Grades 5 to 8, covering menstruation, sanitation, and cultural traditions [18]. Menstruation Matters is a website which explains periods and alleviation of dysmenorrhea [19]. Finally, the Periods Pain & Endometriosis Program PPEP Talk^®^ facilitators provide a one-hour presentation for Grade 10 students focusing on dysmenorrhea [20]. These initiatives are similar to those in a recent systematic literature review which examined 16 school-based menstrual health interventions [21]. It found that interventions tended to address single-issue problems and, with the exception of two studies [22,23], the skills to personally apply OM cycle science were not taught. The review recommended that future programs use comprehensive and strengths-based teaching of the OM cycle and that schools engage externally with healthcare providers and parents [21].

A formative research study was subsequently initiated [24]. By adopting the recommendations of the literature review [21], My Vital Cycles^®^ distinguished itself from the above initiatives. Firstly, it addressed multiple OM problems, which, secondly, was done by giving specific instructions on how to personally apply OM cycle science. Thirdly, its lessons were comprehensive because they prioritized ovulation. Lessons were also based on Nutbeam’s Health Outcome Model [25], which is used in the Western Australian Health and Physical Education (HPE) curriculum [26] to measure the sequential acquisition of health literacies beginning with basic knowledge discovery (functional health literacy); progressing to personal application and communication skills (interactive health literacy); and culminating in social awareness and capability of appraising information (critical health literacy) [25]. Furthermore, its strengths-based teaching was predicated on the Whole Person so that science instruction and skills’ development were located within the girls’ social and emotional contexts. Finally, its whole-school approach adopted the WHO’s Health Promoting School (HPS) framework, which ensured the inclusion of parents and community healthcare providers [27].

The study’s ontology was critical realism, which accepted the reality of OM health literacy and recognized that knowledge of it is limited and subjective [28]. Epistemologically, the study’s formative research sought to explore the mechanisms driving OM health literacy [28]. The methodology was realist inquiry, which is considered a pragmatic approach to address a research purpose [28], specifically that of improving poor OM health literacy. This justified the use of a two-phase sequential mixed-methods protocol [24] as follows:

1. Quantitative Phase: a quasi-experimental pre–post evaluation aimed to test the hypothesis that girls’ OM health literacy is associated with participating in the intervention program (namely, My Vital Cycles^®^);
2. Qualitative Phase: post-program focus group discussions (FGDs) aimed to explore participants’ perceptions of My Vital Cycles^®^ and to gather recommendations for its refinement.

The authors use terms such as females, girls, and women in relation to sex (i.e., biological characteristics or reproductive organs). This may differ from gender identity. The authors believe anyone with OM cycles should have the health literacy needed for them. 

## 2. Materials and Methods

### 2.1. Study Design Overview

The research plan employed a mixed-methods single-arm quasi-experimental design. This started with the Quantitative Phase of a pre–post single group evaluation using the adolescent OM health literacy questionnaire [3]. The intervention was My Vital Cycles^®^, described below in Section 2.3. The Qualitative Phase of FGDs began post-program. The study’s duration from recruitment to final data collection was 28 weeks. It was conducted once only in one single-sex metropolitan school in Western Australia.

The study was prospectively registered with the Australian New Zealand Clinical Trials Register (ACTRN12619000031167). Ethical approval was received from Curtin University’s Human Research Ethics Committee (HRE2018-0101).

### 2.2. Participants and Setting

Sample size calculations referred to a similar intervention study [29]. To detect a medium-sized difference of four points between pre- and post-program OM health literacy scores at 5% significance and 80% power, a sample size of 105 participants was calculated with a 60% retention rate of 63 participants post-measurement.

Grade 9 was selected as the most appropriate grade because its HPE curriculum accommodated the program’s lessons. Secondly, the earliest age to discern possible ovulatory cycles of one-year post-menarche [30] occur in this year, given the average Western Australian menarcheal age of 12.7 years [31]. Therefore, the inclusion criterion was the entire Grade 9 cohort. The school’s Head of Health advised parents that the program constituted part of the HPE curriculum [26], which meant that every Grade 9 student, including premenarcheal girls, received the program. Parents and students consented to participating in the research component of the study.

The exclusion criteria were students who were not enrolled in the school; who were below Grade 9, because they were less likely to experience ovulatory cycles; or were above Grade 9, because their curricula did not offer suitable opportunities.

**Figure 1:**
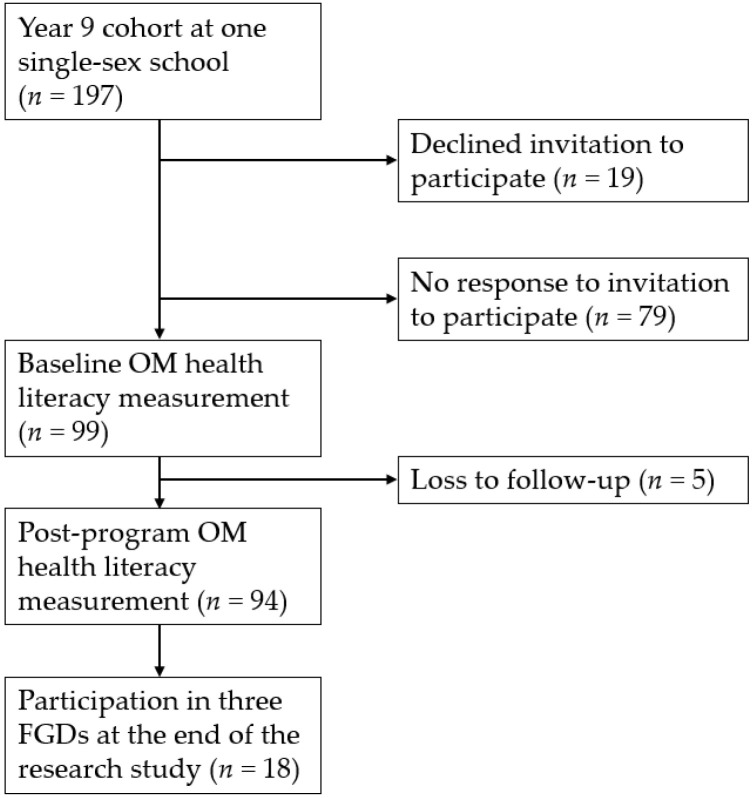
Flowchart of recruitment and retention of participants.

Three information sessions for parents were offered at the school: early morning, late afternoon, and evening. Participant Information Statements were distributed to students as hard copies by teachers who then collected and forwarded the signed consent forms. Electronic copies were also emailed to parents by the Head of Health with a Qualtrics^®^ link for them to consent to their daughter’s participation in the research component. Figure 1 describes the recruitment and retention of participants. Participants had a mean age of 14.46 ± 0.32 years (range 13.92 to 15.25 years).

### 2.3. Piloted Intervention of My Vital Cycles^®^

A Delphi panel of 35 experts in health and education had informed the content of a draft of My Vital Cycles^®^ [32]. This was subsequently reviewed by 28 girls, 5 mothers [33], and 20 school professionals including teachers, nurses, and psychologists. FR, a fertility awareness educator with accreditation recognized by the Australasian Institute for Restorative Reproductive Medicine, facilitated all lessons and consultations, apart from one lesson conducted by medical students under FR’s supervision. My Vital Cycles^®^ observed three Global Standards of the WHO’s HPS framework [27] as detailed below.

#### 2.3.1. School Curriculum (HPS Global Standard 5)

The participating school selected six lessons from the nine provided in My Vital Cycles^®^ (Table 1). These included one school event in the evening for parents and daughters and five 50-min lessons as part of the Grade 9 Health & Physical Education (HPE) curriculum [26]. My Vital Cycles^®^ was conducted over 16 weeks, from 17 May to 3 September 2021 but paused for a four-week term break during this period. The cohort was divided into nine classes, each receiving a lesson approximately every fortnight.

At the start of the program, all students received a Student Journal. The HPE teachers each received a Teacher Guidebook and observed each lesson. The healthcare team of nurses and psychologists each received a Healthcare Professional Guidebook and the timetable of lessons, which they could observe as their schedules allowed.

#### 2.3.2. School Health Services (HPS Global Standard 8)

Individual consultations were offered on campus in the nurses’ clinic for girls to refine their skills of OM cycle observations and interpretation. These were available as a lunchtime drop-in on Wednesdays and on Thursdays after school until 7 pm for the duration of the program. Nurses were invited to observe these consultations as their schedules allowed.

#### 2.3.3. School and Community Partnerships (HPS Global Standard 4)

Parents were invited to attend Lesson 2. At each lesson, students were encouraged to maintain an on-going engagement with their parents at home.

Medical students from three Western Australian Medical Schools were recruited and trained by FR to deliver Lesson 9 as peer-based teaching. Their training was based on the content of My Vital Cycles^®^ to ensure fidelity to the program and to enhance their group facilitation skills. They were not trained to provide clinical OM health advice [34].

### 2.4. Data Collection

#### 2.4.1. Quantitative Phase

OM health literacy was measured as functional, interactive, and critical health literacies [25] with an OM health literacy questionnaire [3] pre- and post-program. Thirty-five experts in health and education provided content validity [32]. It was face validated by 28 girls and demonstrated adequate reliability when test–retested over a two-week period across four school sites with 89 girls [3].

The questionnaire was administered via two online Qualtrics^®^ links. Teachers emailed the pre-program questionnaire link before the first lesson for participants to complete in their own time. The post-program questionnaire link was similarly distributed approximately two weeks after the last lesson. Teachers gave consenting participants time in class to ensure completion.

#### 2.4.2. Qualitative Phase

An open-ended question was included in the post-program questionnaire. Participants were invited to reflect on their experience of My Vital Cycles^®^, suggest improvements, or share insights.

Three focus group discussions (FGDs) of 35 min were facilitated at the end of the Quantitative Phase to explore participants’ perceptions of My Vital Cycles^®^ and to suggest its future refinements. Using the COREQ guidelines [35], this study used the principles of naturalistic inquiry [36] to elicit participants’ experiences. The FGDs were conducted face-to-face in a quiet meeting room at the school. FR welcomed the participants; assured them of their anonymity; and maintained a neutral body position and tone of voice. The FGDs were audio-recorded. These were transcribed by a reputable agency.

The FGD questions are presented in Table 2. They were determined by the study’s realist inquiry and sourced from relevant qualitative literature [37] and intervention studies in health promotion [38]. The final questions were analyzed for their suitability by FR, JH, and SB based on topic relevance; participants’ maturity; and adherence to the study’s realist inquiry’s objective to explore improvements in OM health literacy. The questions were used as a flexible guide, providing structure to the participants’ natural conversation flow by using open-ended questions with follow-up probe questions.

### 2.5. Data Analysis

#### 2.5.1. Quantitative Phase

Normality of the continuous demographic variables of ages was assessed using histogram, boxplot, normal Q–Q plot, skewness, and kurtosis coefficients. Means and standard deviations were used to describe continuous demographic variables because they were normally distributed. Questionnaire responses were dichotomized into either agree (strongly agree/agree) and disagree (neither agree nor disagree/disagree/strongly disagree) or correct and incorrect as appropriate. Cross-tabulation and chi-square analyses or Fisher’s exact tests were performed as appropriate to assess the association between OM health literacy responses and pre- and post-program participation. Significance was set at <0.05. IBM^®^ SPSS^®^ Version 28.0 was used for statistical analyses.

#### 2.5.2. Qualitative Phase

The qualitative descriptive approach used was reflective thematic analysis, which was in keeping with the study’s realist inquiry methodology [28,37]. The flexibility of reflective thematic analysis provided opportunity for inductively developed analysis and enabled descriptive and interpretative accounts of the data [39]. Thematic analysis involved searching and identifying common threads which extended across the open-ended responses from the post-program OM health literacy questionnaire and the FGDs’ transcripts.

Analysis began with familiarization of the data. Audio-recordings were transcribed verbatim and reread with the recordings to ensure accuracy. The open-ended post-program questionnaire responses were downloaded from Qualtrics^®^ into Microsoft Word and categorized.

The clean transcripts and open-ended responses were imported into QSR International NVivo^®^ Release 1.4(4). Preliminary coding expanded as the open-ended responses and transcripts were systematically coded line-by-line in two rounds. Data relevant to each code was collated, then printed in hard copy with succinct labels and broad descriptions to guide the research team’s reflection. Similar codes were then grouped into meaningful patterns and checked against the dataset to determine if they addressed the research question of improving OM health literacy. Further analysis of these patterns facilitated defining and naming of themes and subthemes [40].

Data dependability was maintained by early attention to accurate transcription and on-going reference to the transcripts, which facilitated final refinement of the themes and subthemes. To minimize bias, FR continuously evaluated and reflected on her role within the study and gave regular commentary to reflect on key areas of interests, the participants’ language, and interactions [41]. Bias and confirmability were further addressed by constant comparative data analysis by the research team, which facilitated understanding and interpretation [42].

## 3. Results

### 3.1. Participant Characteristics

Figure 1 describes the outcome of recruitment efforts and the retention of participants after the program delivery. From a cohort of 197 girls, 99 consented to participate, with five lost to follow-up, resulting in a total of 94 participants. The retention rate was 95%.

At pre-program, participants had a mean age of 14.46 ± 0.32 years. At pre- and post-program, their average gynecological ages, measured from reported menarche to date, were 1.55 ± 1.06 years and 1.58 ± 1.10 years, respectively. There were 13 premenarcheal girls.

### 3.2. Quantitative Phase

OM health literacy was measured as functional, interactive, and critical health literacies [19]. Premenarcheal girls were excluded from calculations for questions that required menstrual experience.

#### 3.2.1. Functional OM Health Literacy

Table 3 presents the improvement of basic scientific knowledge of anatomy, physiology, and normal ranges of OM cycle experience post-program for 15 out of 20 items (*p* < 0.05). No difference was observed post-program for the participants’ opinion on the enjoyment of finding OM cycle information (*p* = 0.072), including the understanding (*p* = 0.587), cross-checking (*p* = 0.056), or discernment of its trustworthiness (*p* = 0.142).

#### 3.2.2. Interactive OM Health Literacy

Overall, interactive OM health literacy improved for 19 out of 53 items (*p* < 0.05). Table 4 shows the measures of how information is applied personally.

Pre- and post-program results remained similar for tracking the OM cycle; trusted sources of OM information (except for doctors and school nurses); exchanges of questions and answers; cycle concerns; and absenteeism. Post-program, there was greater satisfaction with answers for OM questions (*p* = 0.024), and 17% reported reduced concerns for bleeding quantity (*p* = 0.006).

The improvement in mood disturbance concerns (*p* = 0.002) was a surprising result. Lesson 8 (Table 1) provided evidence-based remedies for OM cycle difficulties such as period pain and mood disturbances. However, the timing of the post-program questionnaire meant that there were insufficient cycles to measure alleviation of these difficulties.

In addition, post-program improvements (*p* < 0.05) were observed for descriptions of personal OM cycle experiences (*p* = 0.001); self-care (*p* = 0.038) including remedies for OM cycle concerns (*p* < 0.001); goal setting for OM cycle health (*p* = 0.007); and confidence in engaging proactively with a healthcare provider to restore OM cycle health (*p* < 0.001).

#### 3.2.3. Critical OM Health Literacy

Table 5 presents the post-program improvement in critical OM health literacy for 18 out of 25 items (*p* < 0.05), resulting in a 10.3–23.8% agreement range on the usefulness of the OM cycle for self-understanding (*p* = 0.001); holistic health management (*p* = 0.002); beliefs about normality of OM cycle dysfunctions (*p* < 0.001); and lifestyle impacts on fertility. Correct answers for applying OM cycle knowledge to identify OM cycle events in the case studies also improved (*p* < 0.001).

Instances of low improvement were observed on the usefulness of the OM cycle for planning and determining pregnancy; menarche as a milestone; women’s dislike of their periods; and the impact on fertility from undiagnosed OM cycle dysfunctions, and the consumption of drugs, cigarettes, and alcohol.

### 3.3. Qualitative Phase

There were 52 anonymous reflections from the open-ended post-program questionnaire. Eighteen girls attended three FGDs. Table 6 shows the four themes and ten subthemes with illustrative quotes, which describe these participants’ experiences of the program. Pseudonyms replaced actual names.

#### 3.3.1. Increasing Comfort Levels

Some girls indicated an initial hesitancy to engage with the program, which improved in some instances over its duration, as illustrated by this anonymous feedback: “Some of us, including I must confess me, had a bad attitude towards it and felt uncomfortable at first. But I think it’s a great program with a great message, and I admire what you’re doing for young women.” [Anon].

This suggests that the personal topic of menstruation left the girls uncomfortable. By the end of the program, girls expressed improved comfort and confidence around this common experience, for example, “I am more comfortable with people in my classes now. Like before, if I had to go to the bathroom to change a pad or something, I would go up to the teacher privately. Now I’d do the same, but I wouldn’t really care as much if someone overheard.” [Saskia].

#### 3.3.2. Informative

There was strong agreement that the information was useful and helpful, which lead Luna to assert that “I reckon you can understand yourself better now”. Scarlett elaborated further with “talking to my friends in other schools, they knew not even half as much stuff that I’ve learnt”. This was similarly echoed by Maggie, “Even though this school did try to educate us in earlier years, there was a lot of stuff that I didn’t actually realize were important that actually are. And I like it.” [Maggie].

Comments indicated that the potentially dry science was presented in a realistic way with which the girls could identify. As Aurora explained, “It showed that different people go through different stuff and that every period is different, and it kind of let it be a bit more like relatable to us.” [Aurora].

#### 3.3.3. Including Non-Teaching Support

The program’s compliance with the WHO’s HPS Global standards 4 (for community partnerships) and 8 (for school health services) [24] was demonstrated by actively engaging with parents, medical students, and the school’s healthcare professionals.

For example, parents were central to Lesson 2 (Table 1). Most supported this as “a really good idea” [Iris]. Barriers to parents’ involvement included difficulties in attending because “they work quite a bit” [Iris]. Parental importance, however, was recognized, as Scarlett explained, “And because obviously we’re teenagers, we don’t sometimes want to talk to our parents about school stuff. So maybe really enforcing and encouraging the idea of communication to our parents, a lot.” [Scarlett].

Medical students were included as peer-based teaching in Lesson 9 (Table 1). The majority of participants described this as “the best part” [Anon], for example, “I liked how they understood what you were saying even if you weren’t saying it right, you know? It made you feel like you were understanding more, like if you went to a doctor.” [Ember].

When asked about the low attendance at the school nurses’ clinic, responses included discomfort, lack of time, and forgetfulness. It was perceived as a point of addressing a health need rather than an opportunity for deepening self-awareness, as Maggie clarified, “Girls at our age can feel like they can’t be bothered going unless you were actually having troubles with your period.” [Maggie].

#### 3.3.4. Future Considerations

The program ended positively with most girls suggesting its broader implementation, which evoked Ruby’s reaction, “it would be revolutionary, like how many people it would help would be like just insane.” [Ruby]. This broader reach applied within the school curricula with suggestions of also delivering the program in science, as Luna reasoned, “I reckon you do need to have the science if you’re gonna try and chart because a lot of people don’t understand the cycle even if they think they do.” [Luna].

Suggested subjects to refine the program included the hymen, fertility, menopause, and effects of hormonal contraception and COVID vaccines. Finally, the development of an app was recommended because of its convenience, accessibility, and compensation of poor memory.

## 4. Discussion

### 4.1. Interpretation of Quantitative Phase Results Based on Study’s Aims

This study aimed to test the hypothesis that girls’ OM health literacy is associated with participating in the intervention program. Overall, the results indicate that My Vital Cycles^®^ improved girls’ OM health literacy.

#### 4.1.1. Functional OM Health Literacy

Basic knowledge of female anatomy significantly improved, particularly the ability to distinguish between the vagina and vulva. The program emphasized vulval functions as central to the observational skills for recognizing menstruation and likely ovulation [43] and combined this with fundamental knowledge of normal OM cycle experience. Without functional OM health literacy [25], the subsequent skills of interactive OM health literacy [25] to identify then determine the health of personal OM cycle phases are challenged [10,13,43].

#### 4.1.2. Interactive OM Health Literacy

The improvements in self-reported personal knowledge of Day 1 and likely ovulation indicate that development of these skillsets had begun. This confirms the earlier research of Cabezón and colleagues [22] and Klaus and colleagues [23], which demonstrated that perimenarcheal girls can learn these skillsets [22,23].

Participants’ improved knowledge was matched with an increased confidence to describe and explain cycle patterns to a healthcare provider, which González [13] identified as an important step in seeking help [13]. My Vital Cycles^®^ achieves this by teaching the OM cycle in its entirety by reference to the ovarian continuum [44] and recognizing that ovulation drives the cycle [4]. This enables menses to be distinguished from other bleeds [45,46], which is important for answering doctors’ simple question of ‘when was your last period?’ Combined with an increased awareness of evidence-based remedies for OM cycle difficulties and setting goals, My Vital Cycles^®^ offers a practical possibility for patients and healthcare providers to work proactively together to restore cycle health [10,13,45,47].

Arguably, the surprising result of improvement in mood concerns may exemplify the results of Alleva and colleagues’ study [48] of 81 women of average age 22.77 years whereby focusing on body functionality improves body image, fosters body satisfaction, and enhances body appreciation [48]. Relatedly, the linear regressions of Chrisler and colleagues [49] with 72 women aged 18–45 years predicted that those who appreciated their bodies were more likely to express feelings of positive menstrual wellbeing [49]. It is therefore possible that the Whole Person approach of My Vital Cycles^®^, which focused on body functionality within social and emotional contexts, may account for this surprising result.

#### 4.1.3. Critical OM Health Literacy

The sequential culmination of functional and interactive OM health literacies is realized in the acquisition of critical health literacy [25]. Peralta and colleagues [50] observed that critical health literacy has especially been neglected. They recommended its emphasis if school-based programs aim to enhance the knowledge and skills of young people to ensure they can determine informed daily health choices throughout their future lives [50]. In this study, an improved appreciation of how the OM cycle impacts and is impacted by personal health [51] is demonstrated in the increased understanding that the OM cycle is useful as an overall health monitor [2,4,9,10,45].

This sharply contrasts with 56 of the 67 HPS health interventions included in Langford and colleagues’ review [52], which did not describe any educational impacts [52]. Peralta and Rowling [53] suggest that research driven by health concerns risks underplaying the importance of educational outcomes [53]. Critical thinking and development of OM cycle skills pervaded My Vital Cycles^®^, as evidenced by the results of the three case studies which tested problem-solving capabilities. These are the skills which will be useful in determining OM cycle health throughout the next 40 years [4] of life.

### 4.2. Interpretation of Qualitative Phase Results Based on Study’s Aims

The post-program FGDs aimed to explore participants’ perceptions of My Vital Cycles^®^ and to gather recommendations for its refinement. Overall, this intervention program was positively received.

#### 4.2.1. Theme 1 Increasing Comfort Levels

This intervention program required the girls to take notice of their reproductive function, which is healthy [4] but stigmatized [5,6,7,8]. An initial hesitancy to engage was replaced by easiness and confidence. Groven’s and Zeiler’s qualitative Norwegian study [54] similarly encountered this transformation in a girls’ lifestyle program. Interviews with seven girls post-program theorized that the body is a site of self-becoming which opens up a world of meaning and understanding of the body and its capacities [54]. The reported improvement of comfort may have led to unexpected positive bodily feelings and experiences.

#### 4.2.2. Theme 2 Informative

Roux and colleagues [33] reported criticisms from 28 girls of average age 16 years on current menstrual health education in Western Australia as a one-size-fits-all depiction of the OM cycle [33]. In contrast, My Vital Cycles^®^ built competencies using relatable science to recognize personal patterns and to determine if these fit within healthy ranges. This follows Wilding’s and Griffey’s call for a personalized approach [55] as well as Groven’s and Zeiler’s emphasis of individual agency [54].

The program’s strengths-based approach intentionally emphasized the OM cycle as an innate positive sign of good health [4], whilst addressing OM cycle dysfunctions pragmatically and optimistically. This accords with the stance of positive psychology advocates Noble and McGrath [56] of seeking understanding through both success and challenge [56]. The program’s teaching accepted managing health struggles throughout life because imperfect OM cycle health is likely, if not now, then at some point over a long reproductive lifespan [4].

#### 4.2.3. Theme 3 including Non-Teaching Support

Peralta and Rowling [53] asserted that if education aims to develop health literacy, then it is necessary to recognize that learning occurs within a broader school context [53]. However, reflections on Langford and colleagues’ review [52,57] found engagement with family and community to be the weakest in HPS interventions [57]. Lahme and colleagues’ qualitative study with 51 Zambian young women aged 13–20 years concluded that HPS could facilitate the creation of a safe environment for them to manage periods [58]. My Vital Cycles^®^ encouraged supportive relationships between the girls, parents, and healthcare professionals internal and external to the school because it intentionally observed the WHO’s HPS framework [27].

Relatedly, Raniti and colleagues’ systematic review [59] of 36 studies revealed a significant protective relationship between higher levels of school connectedness (as promoted with HPS) and depressive and/or anxiety symptoms. They suggested this may be a novel target for supporting mental health [59]. My Vital Cycles^®^ recognized the OM cycle as a biopsychosocial process [5] by following the HPS framework [27], which ensured the program was embedded in a whole-school approach. This may have contributed to the surprising result of improvement in mood concerns.

#### 4.2.4. Theme 4 Future Considerations

Peralta and colleagues [50] highlighted that health literacy results from a dynamic learning process rather than limited short-term educational interventions occurring at a moment in time [50]. Three lessons from My Vital Cycles^®^ were omitted from this trial (see Table 1). Participants suggested additional OM cycle information delivered across science and health curricula as opposed to “a one-off thing” especially because cycles are “a big part of our life”. Additional refinements included developing an app, which may facilitate a broader implementation. It remains to be seen whether these findings support the call for better menstrual health education [16,17].

### 4.3. Interpretation of Results from Other Studies

Evaluation findings are available for two menstrual health education programs currently offered in Australia, namely Menstruation Matters [19] and PPEP Talk^®^ [20], which is derived from the New Zealand Menstrual Education program [60].

The web-based resource Menstruation Matters was developed with four medical doctors, three education professionals, and five young women, then face validated with four young women [19]. Its feasibility study was based on 56 14–25-year-old participants and its encouraging results include 48% of participants changing their perception of a ‘normal’ period and 84% seeking medical attention [19]. The study used the Health Literacy Questionnaire [61], which relies on subjective assessment of general health literacy [61], and the Health Education Impact Questionnaire [62], which evaluates programs educating patients around chronic disease management [62]. Both questionnaires were validated with patients of mean age 65 years [61] and 61 years [62] respectively, experiencing osteoarthritis, joint replacement surgery, cardiac rehabilitation, or chronic obstructive pulmonary disease [61,62]. Although the study did not indicate if these questionnaires had been validated for female adolescents, the Period ImPact and Pain Assessment self-screening tool for teenagers was used [63].

In contrast, My Vital Cycles^®^ was extensively developed [32] and face validated [33], and adolescents’ OM health knowledge and critical analysis skills were age- and sex-appropriately assessed [3]. This study had a larger sample size of 13–15-year-olds, which avoided conflating the developmental stage of a 14-year-old girl with that of a 25-year-old woman.

The Menstrual Education program was developed by Endometriosis New Zealand and has been delivered in schools since 1997 [60]. Its evaluation study with 2643 adolescents aged 14–18 years offered strongly suggestive evidence that its program increased awareness of endometriosis and promoted earlier presentations to specialist healthcare [60]. However, the evaluation tools were changed across years, which makes direct measured comparison series impossible [60].

In contrast, My Vital Cycles^®^ measured OM health literacy as an educational outcome, taught beyond the single issue of dysmenorrhea, involved internal and external healthcare professionals, and engaged with parents.

### 4.4. Limitations and Strengths

This study lacked a separate control group. Although only one physical group existed experimentally, the control group was compiled historically from its own baseline data. This study was conducted in one single-sex independent school. Generalizability cannot be assumed for different schools or across international or cultural contexts. Only one post-program evaluation was conducted. Longer-term knowledge retention and application in the adolescents’ daily lives remain unknown. Nevertheless, this study’s strength is that the program was co-designed with the relevant stakeholders to address a genuine need in OM health literacy. Furthermore, greater attrition had been estimated in the sample size calculation than the outcome of recruitment and retention.

### 4.5. Future Research

Further investigation is warranted to understand the impact of the program on mood and mental health more generally, with more follow-up points post-program to illustrate longer-term knowledge retention and application. Additional research includes trialing a refined My Vital Cycles^®^ in a co-educational or tertiary setting and with different populations, such as culturally and linguistically diverse women.

## 5. Conclusions

This formative research study has given strong suggestive evidence that My Vital Cycles^®^ improves adolescent girls’ ovulatory menstrual health literacy and is positively regarded. This strengths-based program improved self-understanding, agency, and school connectedness, which may account for the unexpected improvement in mood concerns.

## Figures and Tables

**Table 1:** My Vital Cycles^®^ Health & Physical Education curriculum-mapped program.

Lesson	Location	Content	Health Literacy ^†^	Lesson Trialed
1	Home based	Genealogy OM cycle: a personal health monitor	F I	
2	School hosted family event	Rites of passage Cultural beliefs	I C	*
3	In class	Typical OM cycle overview	F	*
4	In class	Charting skills	F I	*
5	In class	Common OM dysfunctions	F I	*
6	Home based	Critique of misinformation	C	
7	In class	Menstrual stigma	I C	
8	In class	Remedies for OM dysfunctions	F I	*
9	In class	Communication skills	I	*

^†^ F = Functional|I = Interactive|C = Critical [19]. * Indicates selected by the participating school.

**Table 2:** Focus Group Discussion questions and follow-up probe questions.

Please share your experiences of participating in My Vital Cycles^®^
Question One: What do you think about the program?
Follow-up probe questions:
- What is your overall impression about the program? - How did it help you understand how your body works? - What’s it like talking now about cycle functions in general?
Question Two: What changes would improve your experience of the program?
Follow-up probe questions:
- Would there be any topics you would add or take away? - What changes do you suggest for how the program was run? - How did it help your learning by including people other than teachers (e.g., the medical students)? - How were your health and wellbeing supported by including the school’s healthcare team in the program?

**Table 3:** Functional OM health literacy results from My Vital Cycles^®^ evaluation study.

	Items	Pre-Program % (*n*)	Post-Program % (*n*)	*p*-Value
Q3.1	Identification of cervix ^†^	64.6% (64)	85.1% (80)	0.001
Q3.2	Identification of vulva ^†^	39.4% (39)	73.4% (69)	<0.001
Q3.3	Identification of ovary ^†^	86.9% (86)	97.9% (92)	0.004
Q3.4	Identification of uterus ^†^	69.7% (69)	88.3% (83)	0.002
Q3.5	Identification of vagina ^†^	35.4% (35)	73.4% (69)	<0.001
Q3.6	Identification of Fallopian tubes ^†^	88.9% (88)	95.7% (90)	0.075
Q10.1	Enjoyment of finding information ^^^	43.4% (43)	56.4% (53)	0.072
Q10.2	Comprehension of information found ^^^	67.7% (67)	71.3% (67)	0.587
Q10.3	Discernment of information’s trustworthiness ^^^	34.3% (34)	47.9% (45)	0.056
Q10.4	Cross-checking of information ^^^	31.3% (31)	41.5% (39)	0.142
Q12	Identification of oldest age for menarche ^†^	40.4% (40)	86.2% (81)	<0.001
Q13	Determination of Day 1 ^†^	50.5% (50)	88.3% (83)	<0.001
Q14	Identification of period duration ^†^	26.3% (26)	87.2% (82)	<0.001
Q15	Identification of frequency of changing sanitary wear ^†^	42.4% (42)	72.3% (68)	<0.001
Q16	Identification of longest cycle length ^†^	7.1% (7)	66.0% (62)	<0.001
Q17	Definition of ovulation occurrence ^†^	9.1% (9)	63.8% (60)	<0.001
Q18	Identification of ovum survival ^†^	16.2% (16)	72.3% (68)	<0.001
Q19	Identification of ovum age ^†^	34.3% (34)	90.4% (85)	<0.001
Q20	Identification of average age of pregnancy difficulty ^†^	17.2% (17)	80.9% (76)	<0.001
Q22	Understanding the significance of cervical mucus ^†^	30.3% (30)	68.1% (64)	<0.001

Chi-squared or Fisher’s Exact tests were performed|Significance level (α) was set at 0.05. ^†^ Correctly answered|^ Strongly agreed or agreed.

**Table 4:** Interactive OM health literacy results from My Vital Cycles^®^ evaluation study.

	Items of Strongly Agree or Agree	Pre-Program % (*n*)	Post-Program % (*n*)	*p*-Value
Q8	OM cycle tracking ^†^			
	- Cycle is not tracked	32.6% (28)	25.9% (21)	0.347
	- Using a diary or calendar (paper based)	15.1% (13)	19.8% (16)	0.429
	- Using a mobile application	47.7% (41)	44.4% (36)	0.676
	- Using the Oral Contraceptive Pill	4.7% (4)	8.6% (7)	0.299
Q9	Trusted sources for OM information ^^^			
	- Mother	86.9% (86)	91.5% (86)	0.303
	- Father	6.1% (6)	10.6% (10)	0.249
	- Family older female	11.1% (11)	19.1% (18)	0.118
	- Family older male	1.0% (1)	1.1% (1)	0.971
	- Family peer female	28.3% (28)	24.5% (23)	0.548
	- Family peer male	0.0% (0)	1.1% (1)	0.304
	- Friends	55.6% (55)	56.4% (53)	0.908
	- Doctor	27.3% (27)	42.6% (40)	0.026
	- School nurse	18.2% (18)	31.9% (30)	0.027
	- School teacher	8.1% (8)	14.9% (14)	0.137
	- School counsellor	2.0% (2)	3.2% (3)	0.609
	- Websites	22.2% (22)	14.9% (14)	0.191
	- Social media	11.1% (11)	4.1% (8)	0.544
	- Apps	26.3% (26)	24.5% (23)	0.775
	- Magazines or books	5.1% (5)	8.5% (8)	0.338
Q10.5	Comfortable asking OM related questions	43.4% (43)	50.0% (47)	0.361
Q10.6	Satisfied with answers received	55.6% (55)	71.3% (67)	0.024
Q10.7	Considers how OM information personally applies	57.6% (57)	63.8% (60)	0.374
Q11.1	Have someone to go to about OM cycles	84.8% (84)	89.4% (84)	0.351
Q11.2	Open conversations at home	48.5% (48)	59.6% (56)	0.122
Q26.1	Personal knowledge of OM cycle day ^†^	19.8% (17)	43.2% (35)	0.001
Q26.2	Personal knowledge of likely ovulation ^†^	7.0% (6)	34.6% (28)	<0.001
Q26.3	Ability to predict next menstruation ^†^	39.5% (34)	51.9% (42)	0.110
Q26.4	Ability to describe period duration ^†^	58.1% (50)	65.4% (53)	0.333
Q26.5	Ability to describe period flow ^†^	59.3% (51)	74.1% (60)	0.043
Q26.6	Ability to describe cycle length ^†^	32.6% (28)	46.9% (38)	0.058
Q26.7	Awareness of remedies for OM cycle difficulties ^†^	31.4% (27)	86.4% (70)	<0.001
Q26.8	Ability to explain OM cycle accurately to health carer ^†^	39.5% (34)	59.3% (48)	0.011
Q27.1	Spend time understanding OM cycle ^†^	22.1% (19)	16.0% (13)	0.321
Q27.2	Self-care at different OM cycle stages ^†^	26.7% (23)	42.0% (34)	0.038
Q27.3	Setting goals for healthy eating to support OM health ^†^	18.6% (16)	38.3% (31)	0.005
Q27.4	Setting goals for exercise to support OM health ^†^	27.9% (24)	48.1% (39)	0.007
Q27.5	Setting goals for sleeping to support OM health ^†^	25.6% (22)	45.7% (37)	0.007
Q28	Current OM cycle experiences ^^,†^			
	- No concerns	17.4% (15)	25.0% (20)	0.233
	- Irregular cycle concerns	36.0% (31)	28.7% (23)	0.316
	- Length of bleed concerns	17.4% (15)	12.5% (10)	0.374
	- Quantity of bleed concerns	26.7% (23)	10.0% (8)	0.006
	- Mood disturbance concerns	61.6% (53)	37.5% (30)	0.002
	- Cramps or pain with bleed concerns	61.6% (53)	60.0% (48)	0.830
	- Cramps or pain without bleed concerns	26.7% (23)	23.8% (19)	0.657
	- Nausea concerns	39.5% (34)	28.7% (23)	0.144
Q29	Frequency of missing school from OM cycle concerns ^†^			
	- Not missed any school	74.4% (64)	79.0% (64)	0.483
	- Occasionally (<1 day in last two terms)	12.8% (11)	11.1% (9)	0.738
	- Often (<3 days in last two terms)	5.8% (5)	7.4% (6)	0.678
	- Frequently (<5 days in last two terms)	1.2% (1)	1.2% (1)	0.966
Q30.1	Confidence to describe intensity of OM cycle concerns ^†^	56.0% (47)	84.8% (56)	<0.001
Q30.2	Confidence to describe duration of OM cycle concerns ^†^	51.2% (43)	78.8% (52)	<0.001
Q30.3	Confidence to determine likely ovulation to time tests ^†^	20.2% (17)	57.6% (38)	<0.001
Q30.4	Confidence to determine likely ovulation for treatment ^†^	25.0% (21)	57.6% (38)	<0.001

Chi-squared or Fisher’s Exact tests were performed|Significance level (α) was set at 0.05. ^†^ Postmenarcheal respondents|^ >1 response allowed.

**Table 5:** Critical OM health literacy results from My Vital Cycles^®^ evaluation study.

	Items	Pre-Program % (*n*)	Post-Program % (*n*)	*p*-Value
Q4.1	Usefulness of OM cycle for self-understanding ^^^	60.6% (60)	81.9% (77)	0.001
Q4.2	Usefulness of OM cycle to determine overall health ^^^	78.8% (78)	93.6% (88)	0.003
Q4.3	Usefulness of OM cycle to manage OM concerns ^^^	71.7% (71)	89.4% (84)	0.002
Q4.4	OM cycle gives confidence to manage health ^^^	68.7% (68)	87.2% (82)	0.002
Q4.5	Usefulness of the OM cycle to plan pregnancy ^^^	82.8% (82)	88.3% (83)	0.281
Q4.6	Usefulness of the OM cycle to determine pregnancy ^^^	85.9% (85)	85.1% (80)	0.882
Q11.3	Menarche as a milestone ^^^	40.4% (40)	47.9% (45)	0.296
Q11.4	Impression that people agree OM cycles are healthy ^^^	66.7% (66)	83.0% (78)	0.009
Q11.5	Impression that women dislike their periods ^^^	86.9% (86)	79.8% (75)	0.186
Q11.6	Belief that period pain is normal ^^^	88.9% (88)	54.3% (51)	<0.001
Q11.7	Belief that mood swings are normal ^^^	89.9% (89)	67.0% (63)	<0.001
Q21.1	Impact of irregular cycles on fertility ^^^	66.7% (66)	79.8% (75)	0.040
Q21.2	Impact of undiagnosed cycle dysfunction on fertility ^^^	75.8% (75)	86.2% (81)	0.066
Q21.3	Impact of weight on fertility ^^^	45.5% (45)	67.0% (53)	0.003
Q21.4	Impact of poor sleep on fertility ^^^	48.5% (48)	72.3% (68)	<0.001
Q21.5	Impact of stress on fertility ^^^	64.6% (64)	86.2% (81)	<0.001
Q21.6	Impact of drugs or cigarettes on fertility ^^^	79.8% (79)	88.3% (83)	0.108
Q21.7	Impact of alcohol on fertility ^^^	73.7% (73)	84.0% (79)	0.080
Q21.8	Impact of sexually transmitted infections on fertility ^^^	72.7% (72)	88.3% (83)	0.007
Q23.1	Case 1: Identify Day 1 ^†^	53.5% (53)	90.4% (85)	<0.001
Q23.2	Case 1: Determine likely ovulation ^†^	2.0% (2)	55.3% (52)	<0.001
Q24.1	Case 2: Identify Day 1 ^†^	60.6% (60)	92.6% (87)	<0.001
Q24.2	Case 2: Determine likely ovulation ^†^	4.0% (4)	61.7% (58)	<0.001
Q25.1	Case 3: Identify Day 1 ^†^	16.2% (16)	60.6% (57)	<0.001
Q25.2	Case 3: Determine likely ovulation ^†^	18.2% (18)	76.6% (72)	<0.001

Chi-squared or Fisher’s Exact tests were performed|Significance level (α) was set at 0.05. ^†^ Correctly answered|^ Strongly agreed or agreed.

**Table 6:** Themes and subthemes from Focus Group Discussions for My Vital Cycles ^®^.

Theme	Subtheme	Illustrative Quotes
Increasing comfort levels		
	1.1 Initial disengagement	The valuing of the program wasn’t there to just try it out. [Zoe]
	1.2 Comfortable	So talking afterwards, the program made us a lot more comfortable about it, and less stigma around it as well. [Eleanor]
2. Informative		
	2.1 Useful and helpful	It taught us a lot about our cycles and how we can understand if we are healthy or not. [Yasmin]
	2.2 Relatable	There were a lot of different like situations, yeah, that you can relate to your kind of situation in your life. [Willow]
3. Including non-teaching support		
	3.1 Value of parents	Like the parents have still got to help us with this. [Jaylani]
	3.2 Peer-based teaching	It was helpful seeing how professional people talk about it. It made me a lot more comfortable. [Sophia]
	3.3 School nurses’ clinic	It was good knowing it was there. I just never needed it. [Lily]
4. Future considerations		
	4.1 Broader implementation	I believe that it is something that shouldn’t be a one-off thing. It should be a topic that we learn in school especially at all-girls schools as it is a big part of our life. [Anon]
	4.2 Additional information	Maybe do some less commonly known information [Anon]
	4.3 App development	If this program had an app, like I’d definitely use it. [Ruby]

## Data Availability

The re-identifiable data supporting the reported results is not available in accordance with HREC approval.
